# Bcl-2-associated transcription factor 1 Ser290 phosphorylation mediates DNA damage response and regulates radiosensitivity in gastric cancer

**DOI:** 10.1186/s12967-021-03004-z

**Published:** 2021-08-09

**Authors:** Jia Liu, Jingyi Li, Zhao Sun, Yangmiao Duan, Fengqin Wang, Guangwei Wei, Jing-Hua Yang

**Affiliations:** 1 Key Laboratory for Experimental Teratology of the Ministry of Education, Cancer Research Center, and Department of Cell Biology, School of Basic Medical Sciences, Cheeloo College of Medicine, Shandong University, Jinan, 250012 Shandong China; 2 Clinical Systems Biology Laboratories, The First Affiliated Hospital of Zhengzhou University, Zhengzhou, 450001 Henan China; 3 Advanced Medical Research Institute, Cheeloo College of Medicine, Shandong University, Jinan, 250012 Shandong China

**Keywords:** Phosphoproteomics, BCLAF1, Ser290, DNA damage response, Gastric cancer

## Abstract

**Background:**

DNA damage response plays critical roles in tumor pathogenesis and radiotherapy resistance. Protein phosphorylation is a critical mechanism in regulation of DNA damage response; however, the key mediators for radiosensitivity in gastric cancer still needs further exploration.

**Methods:**

A quick label-free phosphoproteomics using high-resolution mass spectrometry and an open search approach was applied to paired tumor and adjacent tissues from five patients with gastric cancer. The dysregulated phosphoproteins were identified and their associated-pathways analyzed using Gene Set Enrichment Analysis (GSEA). The mostly regulated phosphoproteins and their potential functions were validated by the specific antibodies against the phosphorylation sites. Specific protein phosphorylation was further analyzed by functional and clinical approaches.

**Results:**

832 gastric cancer-associated unique phosphorylated sites were identified, among which 25 were up- and 52 down-regulated. Markedly, the dysregulated phosphoproteins were primarily enriched in DNA-damage-response-associated pathways. Particularly, the phosphorylation of Bcl-2-associated transcription factor 1 (BCLAF1) at Ser290 was significantly upregulated in tumor. The upregulation of BCLAF1 Ser290 phosphorylation (pBCLAF1 (Ser290)) in tumor was confirmed by tissue microarray studies and further indicated in association with poor prognosis of gastric cancer patients. Eliminating the phosphorylation of BCLAF1 at Ser290 suppressed gastric cancer (GC) cell proliferation. Upregulation of pBCLAF1 (Ser290) was found in association with irradiation-induced γ-H2AX expression in the nucleus, leading to an increased DNA damage repair response, and a marked inhibition of irradiation-induced cancer cell apoptosis.

**Conclusions:**

The phosphorylation of BCLAF1 at Ser290 is involved in the regulation of DNA damage response, indicating an important target for the resistance of radiotherapy.

**Supplementary Information:**

The online version contains supplementary material available at 10.1186/s12967-021-03004-z.

## Background

GC is one of the most aggressive and therapy-resistant cancers [1]. In China, approximately 405,000 new cases and 325,000 deaths from GC have been reported, making it the second most prevalent disease and the third in cancer-related deaths [2]. Early onset GC is difficult to diagnose due to the histological and genetic heterogeneity of the disease [3]. GC patients are often diagnosed after the disease has progressed to the advanced stage where the long term outlook is very poor and the 5-year survival rate is only 10%-20% [4]. Current treatment strategies for GC include surgical resection, chemotherapy and radiation therapy, however the effects are limited [5, 6]. In recent years, the development of molecular targeted therapy has led to a revolutionary breakthrough and become the hope of cancer treatment. The protein phosphorylation is a critical post-translational modification and therapeutic target in regulating different biological processes [7–9] important for diagnosis, prognosis and treatment of diseases. Thus, a better understanding of GC phosphoproteomics can improve early diagnostic screening and provide effective intervention targets.

Mass spectrometry-based shotgun proteomics has become the leading technology to investigate alteration or modification of proteins [10–12]. It was applied to identify the phosphoproteins in GC, leading to the identification of 162 phosphorylation sites on 49 nonredundant proteins [13]. Notably, Bcl-2-associated transcription factor 1 (BCLAF1), a death-promoting transcriptional repressor highly expressed in a variety types of cancer [14–17], is shown to be phosphorylated at multiple positions. BCLAF1 is involved in a wide range of biological processes including apoptosis, transcriptional regulation and DNA damage repair [18–21]. The BCLAF1 protein contains homologies to the basic zipper and Myb DNA-binding domain and can bind to DNA [22]. Several studies have shown that BCLAF1 plays an important role in DNA damage repair (DDR) [19, 23]. BCLAF1 promotes the transcription of TP53 gene by interaction with PKCδ in response to DNA damage and interacts with γ-H2AX upon ionizing radiation (IR) [24]. BCLAF1 also induces cisplatin resistance in lung cancer cells by regulating DNA damage repair [18]. However, it remained to be determined how BCLAF1 phosphorylation regulates DNA damage response.

In recent years, high-resolution technology in mass spectrometry and better open search algorithms are developed. On this basis, we have developed a quick label-free phosphoproteomics workflow and identified 832 unique phosphorylated sites over 382 proteins from GC, particularly BCLAF1 at Ser290. Bioinformatics analysis showed that the upregulated phosphoproteins were enriched in association with the molecular functions in relevant to DNA damage repair, tumorigenesis and therapy resistance. To address the underlying mechanism, GC cells stably overexpressing BCLAF1 and its mutants were established and their effects on DNA damage repair upon exposure to IR were determined. It was further validated by the clinical data that BCLAF1 Ser290 phosphorylation was upregulated in association with poor prognosis of GC patients.

## Methods

### Tissue samples and cell lines

The protocol for proteomics analysis of the samples was approved by the Institutional Research Ethics Review Boards. Tissue samples were obtained from the tissue band of the National Translational Science Center for Molecular Medicine, Xi’an, China. All tissues were verified by pathologists and stored at − 80 °C. The human AGS and HGC-27 cell lines were obtained from ATCC. HGC cells were cultured in RPMI-1640 medium containing with 10% fetal bovine serum (FBS) and 1% penicillin/streptomycin. AGS cells were cultured in Dulbecco’s modified Eagle’s medium (DMEM) (Gibco) with 10% FBS and 1% penicillin/streptomycin (Gibco). All cells were cultured in a sterile incubator humidified atmosphere containing 5% CO_2_ at 37 °C.

### Sample preparation for LC–MS/MS

Total proteins were extracted from the tumor and adjacent tissue samples using T-PER buffer (Thermo Fisher Scientific) in the presence of protease inhibitor and phosphatase cocktail (Sigma-Aldrich). The concentration of the soluble proteins was determined by Bicinchoninic Acid (BCA) Protein Assay kit (Pierce, thermo scientific, Germany). Equal amounts of the lysates were desalted followed by reduction using 10 mM dithiothreitol and then alkylation using 50 mM iodoacetamide (IAA) in the dark. For digestion, the proteins were mixed with trypsin (Promega) at a protein:trypsin ratio of 25:1 overnight at 37 °C. Peptides were desalted by ZipTip C18 pipette tips (Millipore), washed with 0.1% trifluoroacetic acid (TFA), and eluted with 50% methanol followed by lyophilizing in a SpeedVac for LC–MS/MS analysis. For pre-fractionation, 50 μg of the peptide mix was re-dissolved in 160 μl of ammonia water (pH = 10) and fractionated by high pressure liquid chromatography (Agilent 1100 system, Agilent Technologies Inc., USA) with a reverse-phase C18 column (250 × 0.1 mm, 3 μm Reprosil). The column was eluted with a 60 min-gradient of acetonitrile from 2 to 50% in ammonia water (pH 10.0). A total of 55 fractions were collected, combined into 10 fractions and lyophilized for LC–MS/MS analysis.

### LC–MS/MS analysis

The MS and MS/MS spectra were acquired by an EASY-nLC 1000 system followed by LTQ-Orbitrap Elite mass spectrometer (Thermo Scientific, San Jose, CA) in data-dependent mode. The spray voltage was 2.1 kV and the capillary temperature 275 °C. MS spectra were acquired in the m/z range of 350–1800 at a resolution of 60,000 at 400 m/z. MS/MS fragmentation of the 30 most intense peaks were selected for every full MS scan in the collision-induced dissociation mode. Typically triple technical replicates were analyzed for each fraction.

### Proteomics and phosphorylation analysis

For proteomics, MS/MS spectra were searched against the human protein database using SEQUEST in Proteome Discover. Trypsin was specified as cleavage enzyme allowing up to two missing cleavages. MS/MS spectra were searched with a maximum allowed deviation of 10 ppm for the precursor and 0.6 Da for fragment masses. The oxidation of methionine was selected as dynamic modification, and the false discovery rate (FDR) was 1%. For protein phosphorylation, an open search algorithm was conducted using Byonic, the peptides with the delta mass of 79.96 ± 0.02 Dalton were selected for clustering analysis using Gaussian mixture components. The protein peptides with the expected value ± standard derivation (79.966 ± 0.005) were considered as the identifications of protein phosphorylation, and their spectral counts and intensities used for label-free quantifications.

### Bioinformatics analysis

The up- and down-regulated proteins with phosphorylation (Fold Change or FC > 2, p < 0.05) were identified and subjected to gene ontology (GO) functional annotation analysis. Significant enrichments were determined by the categories under biological process, cell component and molecular function using DAVID Bioinformatics Resources (http://david.abcc.ncifcrf.gov/home.jsp). Enriched pathways were identified from the Kyoto encyclopedia of genes and genomes (KEGG) database. Protein–protein interaction network of the differentially phosphorylated proteins was analyzed using STRING (http://string-db.org/) with the default threshold in the database.

### Immunoprecipitation of the BCLAF1 protein

HGC cell lysates were carried out using RIPA lysis buffer (weak, Beyotime, China) and incubated with 1 µg of the BCLAF1 antibody overnight at 4 °C on a rotating wheel. 30 µL of protein A/G beads (MCE, China) were added to lysates and incubated on a rotator at 4 °C for 2 h. The beads were collected by a magnetic rack, and washed three times with ice-cold PBS-T (0.01% Tween) buffer. Beads were incubated with loading buffer and boiled for 5 min followed by sodium dodecyl sulfate–polyacrylamide gel electrophoresis (SDS-PAGE) and western blot analysis.

### Protein in-gel digestion

The SDS/PAGE gels were cut into small pieces, washed twice with 50 mM NH_4_HCO_3_ buffer at 4 °C for 4 h and then dried with 100% CH_3_CN for 10 min. Sequencing grade modified trypsin (Promega) at a concentration of 10 μg/mL in 50 mM NH_4_HCO_3_ buffer was added for overnight digestion at 37 °C. The peptides were extracted from the gel with 60% CH_3_CN in 0.1% TFA for 30 min. The extracted solution was dried under vacuum in SpeedVac for subsequent mass spectrometry analysis. The obtained data were submitted to Mascot software (Matrix Science) to search for phosphorylated residues on the BCLAF1 protein.

### Specific antibody for phosphorylated BCLAF1 at Ser290

The specific antibody recognizing pBCLAF1 (Ser290) was raised in rabbits against peptide coupling with PSQNS(p)PIH-KLH (corresponding to the residues 286–293 of human BCLAF1). The antibody was prepared and purified by ABclonal Biotech (China). Antibodies used in this study as follow: BCLAF1 (A300-608A) was purchased from Thermo Fisher Scientific, Inc. (USA); γ-H2AX (#80312), Anti-rabbit IgG (H + L, Alexa Fluor 549 Conjugate, #8889), Anti-mouse IgG (H + L, Alexa Fluor 488 Conjugate, #4408), were purchased from Cell Signaling Technology, lnc. (USA); GAPDH (60004–1-Ig), GFP (F1804) were purchased from Proteintech, Ltd. (Shanghai, China).

### Human gastric cancer tissue microarray, immunohistochemistry, and scoring

Human GC tissue microarray (TMA) that consisted of the gastric tumor tissue specimens (n = 95) and adjacent non-normal tissue specimens (n = 85) were purchased from Outdo Biotech, Ltd. (Shanghai, China). The pBCLAF1 (Ser290) antibody was used at a 1:100 dilutions. The assignment of nuclear staining intensity score was based on the staining intensity (no intensity: 0, weak intensity: 1 + , moderate intensity: 2 + , and strong intensity: 3 +) and positive-staining score was based on the percentage of positive-staining (0% positive: 0, 1–25% positive: 1, 26–50% positive: 2, 51–75% positive: 3, and 76–100% positive: 4) by three experienced pathologists. Note that, as the secretion of pepsin and gastric acid from gastric fundic gland could cause false positive signals. the gastric fundic glands were excluded from the interpretation conditions. Plasma cells were not stained and used as the negative control (Additional file 1: Figure S1). The final staining index was calculated using the formula: positive-staining score × staining intensity score. For data analysis, staining scores < 4 were defined as low expression, and scores ≥ 4 indicated high expression.

### Lentiviral plasmid construction and infection

The BCLAF1 lentiviral short hairpin RNA (shRNA) and a negative control vector (NC) were purchased from GeneChem Co., Ltd. (Shanghai, China) and transduced into the HGC and AGS cell lines following the manufacturer’s instructions. The Flag-tagged WT, S290D, and S290A overexpressed lentiviruses were amplified, purified from GeneChem Co., Ltd. (Shanghai, China). The cDNA was subcloned into the GV492 plasmid (Ubi-MCS-3FLAG-CBh-gcGFP-IRES-puromycin) (Genechem) and then co-transfected into HEK293T cells with the lentiviral genomic plasmids. Lentiviral particles were obtained by collecting supernatant using the kit for ultracentrifugation concentration and purification of lentiviral particles. Cells were cultured in 6-well plates until 60% confluent and infected with lentivirus particles at a MOI of 50 in the presence of 10 g/ml polybrene for 48 h. Stable cells were maintained in medium containing 0.5 µg/mL of puromycin (Beijing Solarbio Science & Technology Co., Ltd.).

### MTT assay

Three replicates of equal amounts of cells (3 × 10^3^/well) were seeded into 96-well plates and incubated for various durations. The cells were incubated with 20 µL 3-(4, 5-dimethylthiazol- 2-yl)-2, 5-diphenyltetrazolium bromide (MTT, 5 mg/ml in PBS) for 4 h at 37 °C. Then, 150 µL dimethyl sulfoxide (DMSO) was added to the wells, and the optical density (OD) was detected at 490 nm by a microplate reader.

### Colony formation assay

Exponentially growing cells (5 × 10^2^) were seeded into 6-well plates for 10–14 days to form colonies. For the determination of colony formation, the cells were fixed in 4% polyformaldehyde, stained with 1% crystal violet. Colonies of at least 50 cells were counted. The mean ± SD from three independent experiments was determined.

### 5-Ethynyl-2′-deoxyuridine proliferation assay

The 5-ethynyl-2ʹ-deoxyuridine (EdU) assay was performed with an EdU Kit (Byotime, China). Cells were seeded onto 24-well plates and cultured in 0.5 mL medium for 24 h, and then added 0.5 mL of 10 µM EdU into each well for 2 h. Then, cells were fixed with 4% polyformaldehyde at room temperature for 15 min and subsequently incubated with Apollo staining solution for 30 min. The cell nuclei were stained with Hoechst 33,342 (1:1000 in PBS). The proportion of EdU-positive cells was determined with a Zeiss LSM780 confocal microscope system (Carl Zeiss, Germany). Image J_v1.8.0 (National Institutes of Health, USA) was used to count the number of all cells and proliferating cells.

### Irradiation

Cells were ionize-irradiated (3.5 Gy/min) at room temperature using X-RAD225 OptiMAX Biological Irradiator (Precision X-Ray Inc., USA). Once irradiated, the cells were immediately transferred to the incubator at 37 °C in 5% CO_2_ until the designated harvest time.

### Immunofluorescent staining

Cells grown on coverslips were fixed in 4% paraformaldehyde for 15 min at room temperature and then permeabilized with 0.1% Triton X-100 in PBS for 15 min. The cells were blocked with 5% goat serum for 30 min before incubation with primary antibodies overnight at 4 °C. After washing with PBS, cells were incubated with the secondary antibodies for 1 h at room temperature. The cell nuclei were dyed with Hoechst 33342 (1:1000 in PBS). After a final wash with PBS, the slides were covered with anti-fade mounting medium (Beyotime, China). Microscopic analyses were captured at magnification (200× , 630×) with a Carl Zeiss LSM 780.

### Flow cytometry

For cell apoptosis analysis, 1 × 10^6^ cells were washed twice with PBS and stained in 100 µL binding buffer with 5 µL Annexin V-APC and 10 µL 7-ADD for 20 min in the dark at room temperature. Annexin V/PI staining assays were performed following the manufacturer’s protocol (BD Biosciences, San Jose, CA, USA), Then, an additional 400 µL of binding buffer was added to the cell suspension and the samples were determined by CytoFLEX S flow cytometry (Beckman, USA).

### Western blotting

Cell lysates were extracted using RIPA lysis buffer (Beyotime Biotechnology, China) with protease inhibitor cocktail (APExBIO, China) and phosphatase cocktail (APExBIO, China). The protein concentration was measured by a BCA protein assay kit (Vazyme Biotech Co., Ltd). The proteins were mixed with 5 × loading buffer (EpiZyme, China) and boiled at 95 °C for 5 min followed by separate in 10% SDS-PAGE (EpiZyme, China). The protein bands were electrophoretically transferred onto polyvinylidene difluoride membranes (PVDF, Roche, USA). The PVDF membranes were blocked with 5% BSA (Sangon Biotech) for 1 h at room temperature and incubated overnight with primary antibodies in 1% BSA. After incubation with secondary antibody at a dilution of 1:10,000 for 1 h at room temperature, protein bands were visualized by Odyssey infrared imaging system (Li-CorBioscences, USA).

### Statistical analysis

All quantitative data are represented as mean values of at least 3 independent experiments ± standard deviation (SD). Multiple hypothesis testing based on FDR using the Perseus has been used to analyze the foldchange between cancer with para-cancerous tissues. Differences between groups were analyzed by Student’s t-test for two groups and one-way ANOVA for more than two groups. Survival analysis was performed using Kaplan–Meier method and compared with the log-rank test. Pearson chi-squared test and Fisher’s exact test were used to analyze the relationship between pSer290-BCLAF1 expression and clinical features. Cox proportional hazard model was used to determine factors related to patient survival. P < 0.05 was considered as statistically significant value. All statistical analyses were performed with GraphPad Prism 8.0.2 software.

## Results

### Upregulation of DNA damage response in gastric cancer

**Fig. 1:**
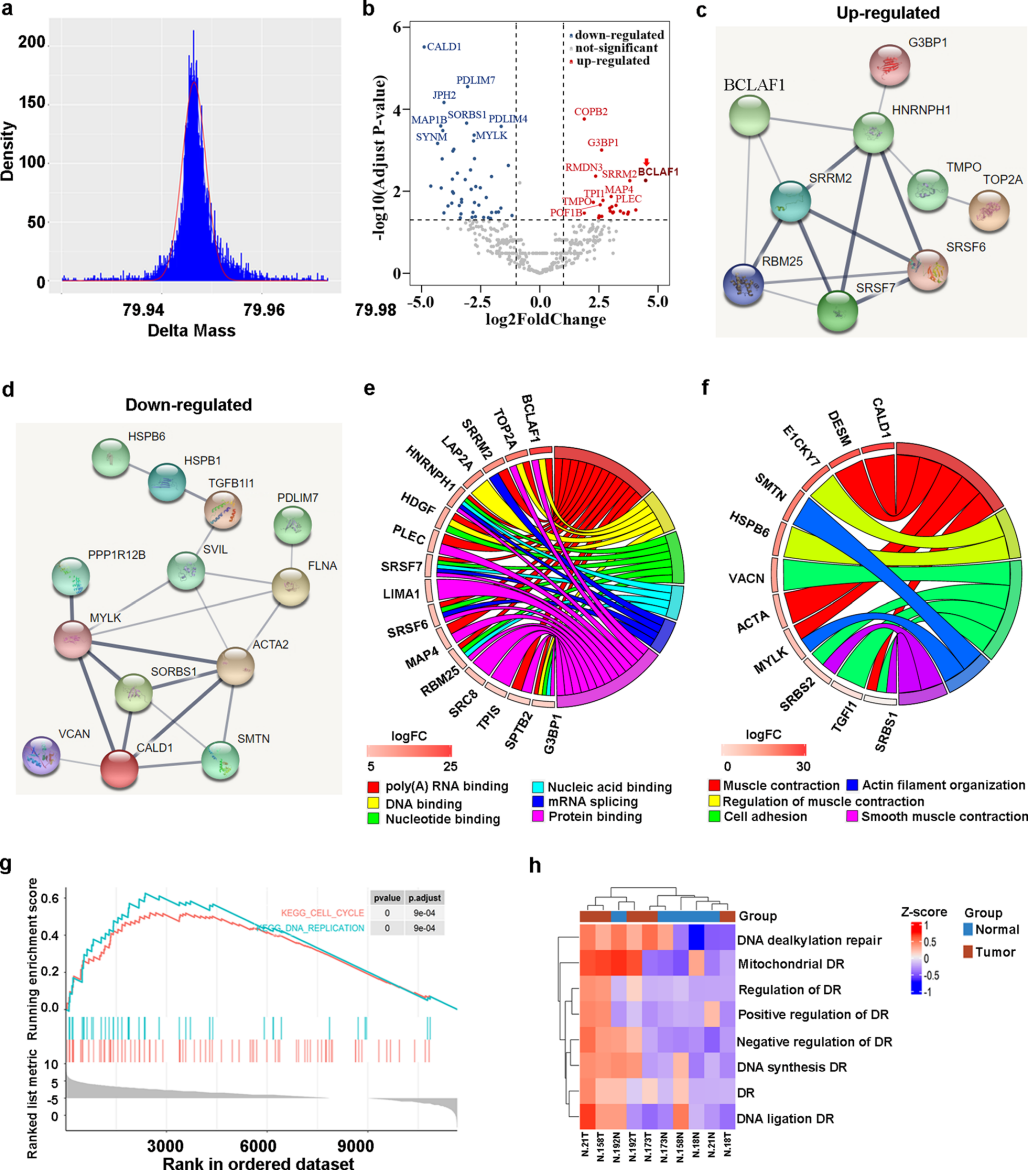
Dysregulated DNA damage response in gastric cancer unraveled by quick label-free phosphoproteomics. **a** Phosphopeptide ratio distribution. **b** Volcano plot to show the differential phosphoproteins with unique phospho-sites in gastric cancer and paired non-tumor tissues, with candidate phosphoproteins marked, including BCLAF1 (dark red). The red points represented the up-regulated phosphoproteins, while blue points represented the down-regulated phosphoproteins. Multiple hypothesis testing based on FDR. **c** PPI of up-regulated phosphoproteins. **d** PPI of down-regulated phosphoproteins. **e** The chord diagram of enrichment analysis for the up-regulated phosphoproteins. **f** The chord diagram of enrichment analysis for the down-regulated phosphoproteins. **g** GSEA analysis of the DEPs between gastric cancer and paired non-tumor tissues. **h** GSVA was used to analyze these DEPs closely related to DNA repair

A modified label-free phospho-proteomic workflow was adapted to quickly identify possible dysregulated protein phosphorylation in GC tissues (Additional file 2: Figure S2). Briefly, the shotgun proteomics was applied to the total proteins from tumor and para-cancerous tissues, and peptide sequences were repeatedly determined by deep fractionation and high-resolution liquid chromatography-tandem mass spectrometry (LC–MS/MS). By allowing single amino acid polymorphisms (SAAPs) in the peptides, the mass differences between the coding amino acids and actual residues were screened using the multi-blind spectral alignment algorithm MODa [25, 26] and Byonic [27]. Nonzero delta masses were clustered within intervals and mass clusters assessed by Gaussian regression to determine the potential protein modifications. Among them, 4243 delta masses were clustered as the well-defined phosphorylation modification (Fig. 1a, + 79.9665 ± 0.0002, n = 4243, R2 = 0.91) that spread over 832 unique phosphorylated sites of 382 different proteins (Additional file 4: Table S1). In comparison of the cancer with para-cancerous tissues, 25 phosphorylated sites were up- and 52 down-regulated (FC > 2, p < 0.05) (Fig. 1b). Notably, the upregulated protein phosphorylations were predominately involved in the interaction with nucleotides, DNAs, and RNAs (Fig. 1c, e), which were apparently associated with dysregulation of DDR, nuclear assembly and genomic instability of GC. Among them, a DDR-associated protein BCLAF1 is the mostly upregulated phosphoprotein. On the other hand, the mostly down-regulated protein phosphorylations were predominately involved in the regulation of muscle contraction, which reflected the function loss associated with GC (Fig. 1d, f). Consistently, GSEA unveiled that differentially expressed proteins (DEPs) in GC were involved in DNA replication (p < 0.0001) and cell cycle (p < 0.0001, Fig. 1g). The functions of these DEPs were predominantly connected with the DNA damage repair pathways, including positive/negative regulation of DNA repair, DNA de-alkylation repair, mitochondrial DNA repair, DNA synthesis, and DNA ligation etc. (Fig. 1h). Taken together, our quick label-free phosphoproteomics suggest that BCLAF1 phosphorylation is upregulated and may be involved in the regulation of the DDR in GC.

### Upregulation of BCLAF1 Ser290 phosphorylation in gastric cancer

**Fig. 2:**
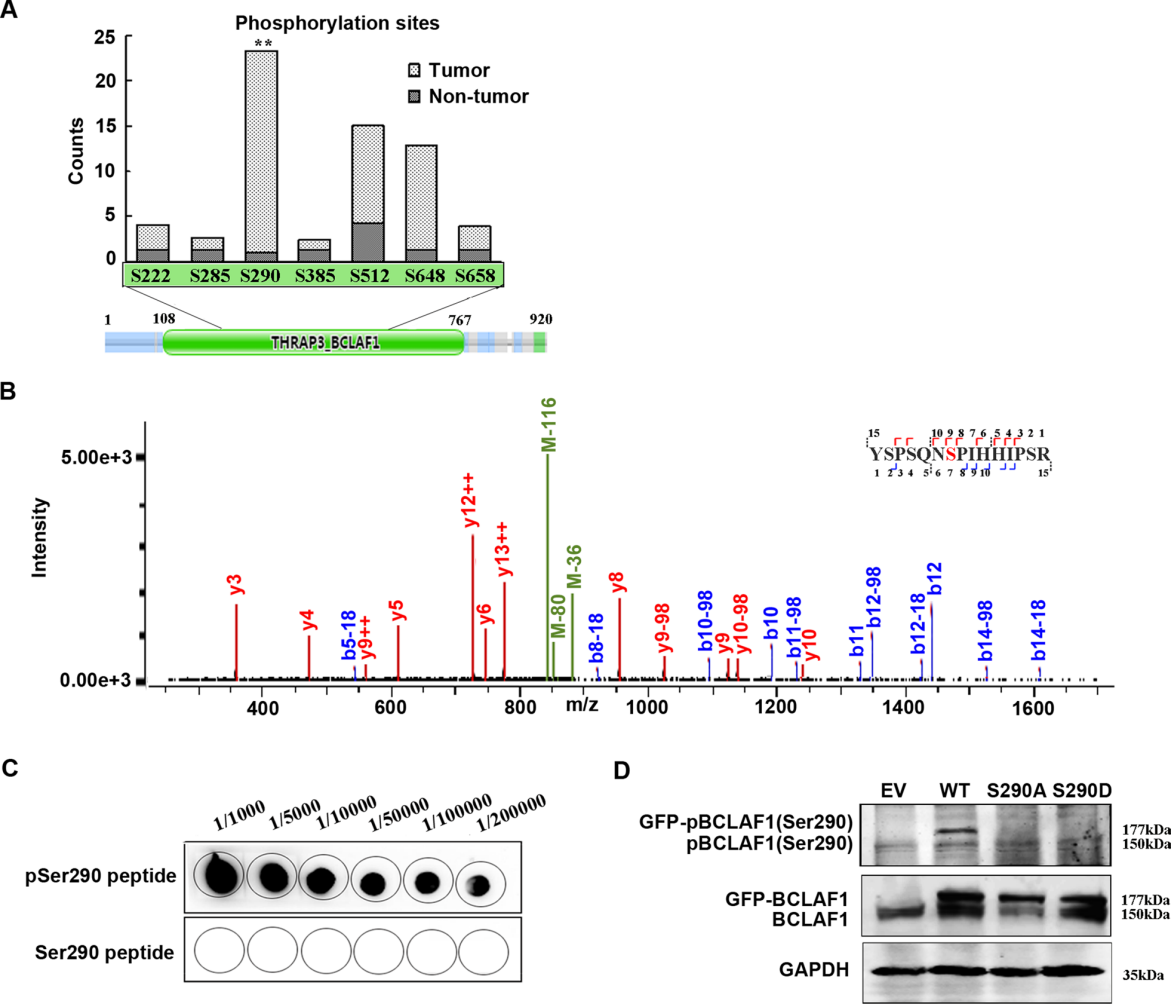
Phosphorylation of BCLAF1 Ser290 is significantly increased in gastric cancer. **a** Total counts of phosphorylated residues in BCLAF1. **p < 0.01 vs. Non-tumor based on Student's t-test. **b** MS/MS spectrum of pSer290-containing peptide, YSPSQN(pS290)PIHHIPSR in HGC gastric cancer cells. **c** Dot blotting of the phosphorylated (pSer290) and unmodified (Ser290) peptides with anti-pBCLAF1 (Ser290) antibody. **d** Western blotting of the total proteins from HGC cells overexpressing WT, S290A and S290D forms of BCLAF. 177 kD, GFP-tagged BCLAF; 150 kD, endogenous BCLAF

Among the 7 phosphorylated residues we identified at BCLAF1, the most significantly upregulated site was Ser290 (FC = 22.3, p = 0.005, n = 18) (Fig. 2a), locating at the THRAP3 domain of BCLAF1 involved in response to DNA damage [21]. To verify the phosphorylation at BCLAF1 Ser290 (pBCLAF1 (Ser290)), total proteins were isolated from HGC-27 (HGC) GC cells and immunoprecipitated with anti-BCLAF1 antibody. The gel area with the positive signal was excised and digested with trypsin. The phosphorylated BCLAF1 peptide, YSPSQN(Sp290)PIHHIPSR, was confirmed by LC–MS/MS analysis (Fig. 2b). To further confirm the phosphorylation of BCLAF1 at Ser290, a synthetic peptide containing the phosphorylated Ser290 was used to generate a specific polyclonal antibody. Dot-blotting assay showed that the antibody specifically recognized the phosphorylated peptide (Fig. 2c). GFP-tagged wild-type BCLAF1 (WT), and BCLAF1 with Ser290A (S290A) or Ser290D (S290D) mutation were transiently overexpressed in HGC cells, the endogenous BCLAF1 and the GFP-tagged BCLAF1 (WT, S290A or S290D) were recognized by the anti-BCLAF1 antibody. The anti-pBCLAF1 (Ser290) antibody detected the endogenous pBCLAF1 and the GFP-tagged WT pBCLAF1 (Fig. 2d); however, no signal was detected in the unphosphorylated BCLAF1 with S290A/D mutants. To further confirm the specificity of the pBCLAF1 (Ser290) antibody, negative controls were added by pretreatment of the lysates with CIP (phosphatase) to remove the phosphorylated form of BCLAF1 before western blotting. As shown in the Additional file 3: Figure S3, the signals for the endogenous pBCLAF1 (Ser290) and transiently expressed one were abolished, supporting that the pBCLAF1 (Ser290) antibody is specific to the phosphorylated proteins.

**Fig. 3:**
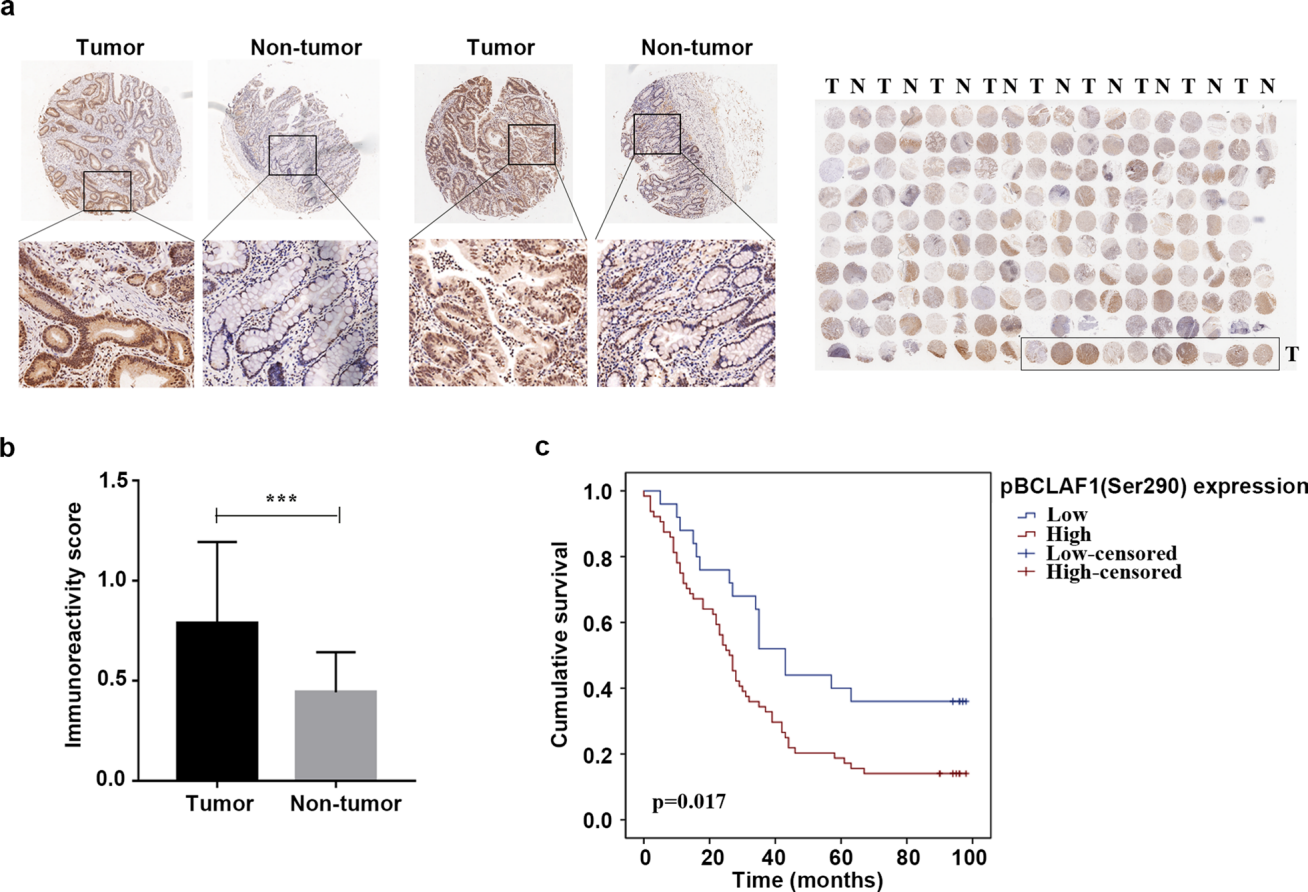
BCLAF1 Ser290 phosphorylation is correlated with gastric cancer development. **a** Immunohistochemistry on gastric tumor tissues and adjacent non-tumor tissues using pBCLAF1 (Ser290) antibody (up-panel 40 × and lower panel 100 × , respectively). **b** Statistical data to show the IHC score of pBCLAF1 (Ser290) staining in the gastric tumor tissues (n = 95) and adjacent non-tumor tissues (n = 85). ***p < 0.001, Student's t-test. **c** Kaplan–Meier survival curve showed that high pBCLAF1 (Ser290) level correlated with poor overall survival of GC patients. p = 0.017, log-rank test

To explore the existence and clinical importance of pBCLAF1 (Ser290) in human GC, the cancer tissue microarray chips were used to perform IHC analysis. pBCLAF1 (Ser290) level was significantly upregulated in gastric tumor tissues compared to matched non-tumor tissues (Fig. 3a). The IHC staining scores were analyzed by the staining index. Statistically, the pBCLAF1 (Ser290) level was found to be significantly higher in the gastric tumor tissues than that in adjacent non-tumor tissues (Fig. 3b), and the high level of pBCLAF1 (Ser290) was positively correlated to poor prognosis (Fig. 3c). The correlation of pBCLAF1 (Ser290) with clinicopathological characteristics in the tissue microarray is further summarized in Table 1, the high pBCLAF1 (Ser290) level was found to be positively correlate with the age and stage of GC patients. Additionally, Univariate analysis revealed that pBCLAF1 (Ser290) levels (p = 0.021), Grade (p = 0.002), T stage (p = 0.002), N stage (p < 0.001) and TNM stage (p < 0.001) were significant prognostic factors for OS. Multivariate analysis further indicated Grade (HR: 1.691; 95% CI: 1.044–2.740; p = 0.033), T stage (HR: 2.161; 95% CI: 1.291–3.617; p = 0.003) and N stage (HR: 1.498; 95% CI: 1.026–2.187; p = 0.036) were shown to be available independent prognostic factors (Table 2). Together, these results confirmed the existence and upregulated level of pBCLAF1 (Ser290) and suggested that the high pBCLAF1 (Ser290) level is correlated to the GC development and poor prognosis in GC patients.

**Table 1:** Correlation between pBCLAF1 (Ser290) expression and clinicopathological characteristics

	Variables	pBCLAF1 (Ser290) expression		Total	χ^**2**^	p value
		Low	High			
Age (year)					4.357	0.037
	<60	11	14	25		
	>=60	14	50	64		
T stage					0.189	0.664
	T1/T2	4	8	12		
	T3/T4	21	56	77		
TNM stage					0.714	0.398
	Ι/II	11	22	33		
	III/IV	14	42	56		
N stage					4.445	0.035
	N0	9	10	19		
	N1/N2/N3	16	54	70		
M stage					2.287	0.130
	M0	23	63	86		
	M1	2	1	3		
Sex					1.420	0.233
	Female	4	18	22		
	Male	21	46	67		
grade					0.084	0.771
	I/II	7	16	23		
	III/IV	18	48	66		

cation of the upregulation of pBCLAF1 (Ser290) suggested a potential biomarker for the prognosis of patients and a possible target to improve radiotherapy sensitivity in GC.

**Table 2:** Univariate and multivariate analyses of the factors correlated with Overall survival of Gastric carcinoma patients

Variables	Univariate analysis			Multivariate analysis		
	HR	95%CI	p value	HR	95%CI	p value
Expression	1.935	1.106-3.386	0.021	1.684	0.958-2.962	0.070
Sex	0.696	0.410-1.183	0.181			
Grade	2.135	1.317-3.462	0.002	1.691	1.044-2.740	0.033
Age	1.016	0.993-1.040	0.166			
T stage	1.805	1.232-2.645	0.002	2.161	1.291-3.617	0.003
N stage	1.484	1.199-2.837	0.000	1.498	1.026-2.187	0.036
M stage	1.594	0.499-5.093	0.431			
TNM stage	2.001	1.377-2.908	0.000	0.878	0.418-1.843	0.731

### BCLAF1 phosphorylation at serine 290 facilitates cell proliferation

**Fig. 4:**
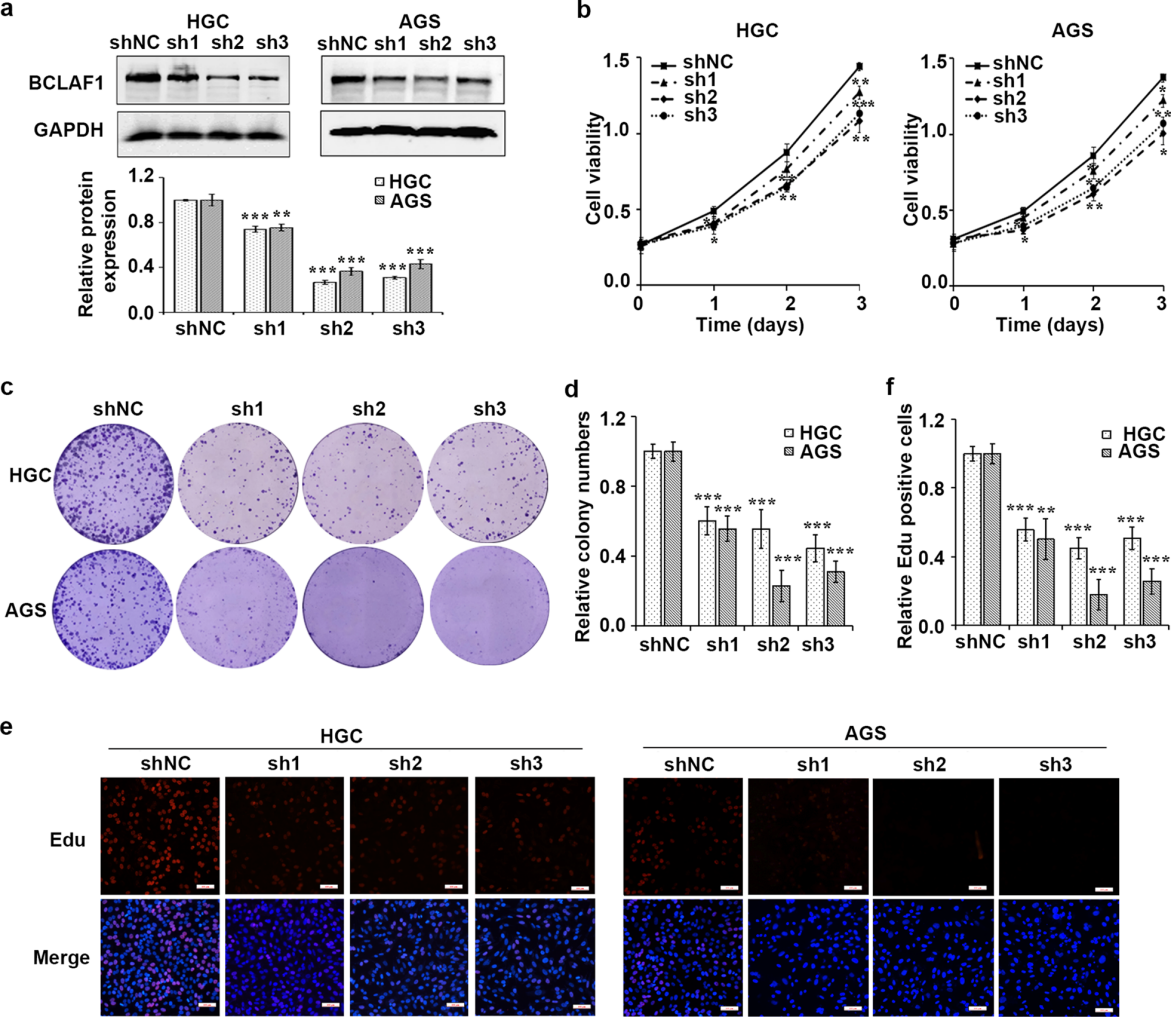
BCLAF1 silence inhibits cell proliferation. **a** Western blot analysis of BCLAF1 expression in HGC and AGS cells infected with shNC or shBCLAF1s (sh1, sh2 and sh3). GAPDH served as an internal reference. **b** The transfected cells were harvested for MTT assay. BCLAF1 depletion inhibited cell proliferation of HGC and AGS gastric cancer cells. **c** Colony formation assay was performed to investigate colony formation ability of shNC or shBCLAF1s cells. **d** Quantitative results of colony formation analyzed with Image J. **e** EdU assay was used to examine the cell proliferation ability of shBCLAF1s transfected cells. Scale bars, 100 µm. **f** Three different fields were randomly chosen and quantitative results of EdU assay analyzed with Image J. Data presented as the mean ± SD of three independent experiments. *p < 0.05, **p < 0.01 and ***p < 0.001 vs. shNC based on Student's t-test

BCLAF1 has been reported to promote cell proliferation and invasion in hepatocellular carcinoma [19], indicating an oncogenic role of BCLAF1 in cancer. Thus, we first assessed the involvement of BCLAF1 in human GC. BCLAF1 deficient cell lines were established by transfecting lentiviral particles encoding shRNA hairpins against BCLAF1 or a nontargeting shRNA (shNC) into human GC cell lines HGC and AGS (Fig. 4a). MTT and colony formation assays indicated that BCLAF1 knockdown delayed cell growth significantly (Fig. 4b–d). Moreover, in vitro EdU incorporation assay showed BCLAF1 silent cells exhibited slower proliferation rate (Fig. 4e, f).

**Fig. 5:**
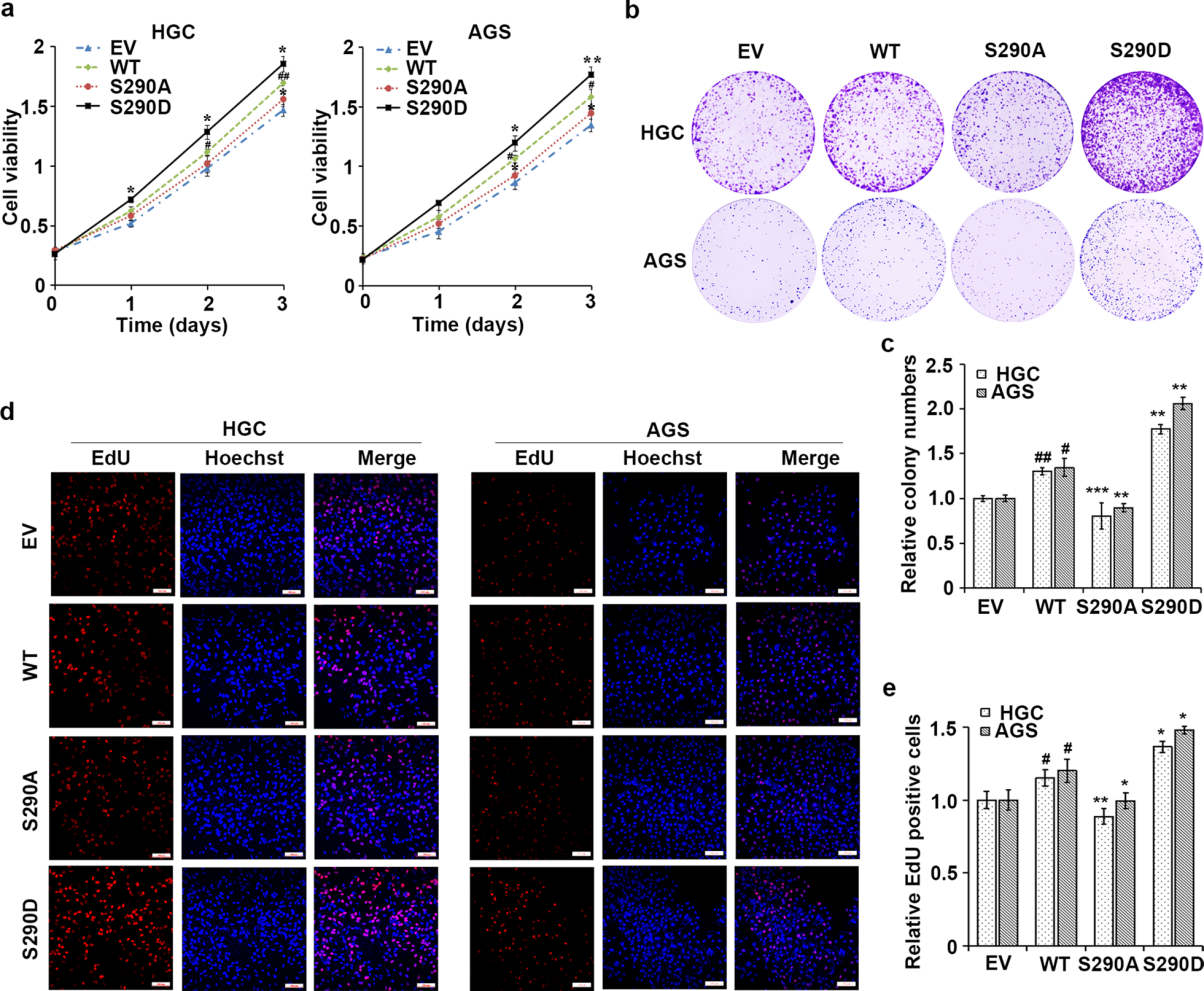
BCLAF1 phosphorylation at Ser290 facilitates cell proliferation. **a** MTT assay of HGC and AGS cells overexpressing BCLAF1-WT (WT), mimic BCLAF1-pSer290 (S290D), nonphosphorylatable Ser290 (S290A) or empty vector (EV). **b** Colony formation assay was performed to investigate colony formation ability in HGC and AGS cells overexpressing WT, S290D, S290A forms of BCLAF1. **c** Quantitative results of colony formation analyzed with Image J. **d** EdU incorporation assay was performed using a fluorescence method in cells transfected with WT, S290D, S290A forms of BCLAF1. Scale bars, 100 µm. **e** For each group, three different fields were randomly chosen and EdU positive cells were counted with Image J. Data are shown as mean ± SD. *p < 0.05, **p < 0.01 and ***p < 0.001 vs. WT. ^#^p < 0.05, ^##^p < 0.01 vs. EV based on Student's t-test

We then investigated if pBCLAF1 (Ser290) affect the oncogenic function of BCLAF1 in GC cells. The cell viability was assessed in GC cells stably expressing WT, S290A, S290D types of BCLAF1 by MTT assay (Fig. 5a). The cell viability of cancer cells transfected with WT BCLAF1 was significantly higher than the cells with empty vector transfection. GC cells with BCLAF1 S290D demonstrated the highest viability at all time points compared with cells transfected with WT and S290A forms of BCLAF1. We then revaluated cell proliferation using colony formation assay and found that WT BCLAF1 enhanced the growth capability of HGC and AGS cells (Fig. 5b, c). Moreover, the number and size of colonies in the cells with BCLAF1 S290D were markedly higher and bigger than in the cells with WT and S290A forms of BCLAF1. Furthermore, using EdU incorporation assay, we found that WT BCLAF1 overexpression was associated with an increased mitotic rate compared to the empty vector cells (Fig. 5d, e). The cell proliferation and mitotic rate in the cells with BCLAF1 S290D was markedly higher than in the cells with WT and S290A forms of BCLAF1. These data indicated that the oncogenic role of BCLAF1 is dependent, at least partially, on the phosphorylation of BCLAF1 at Ser290.

### BCLAF1 Ser290 phosphorylation is involved in DNA damage repair and promotes IR-induced DNA damage response

**Fig. 6:**
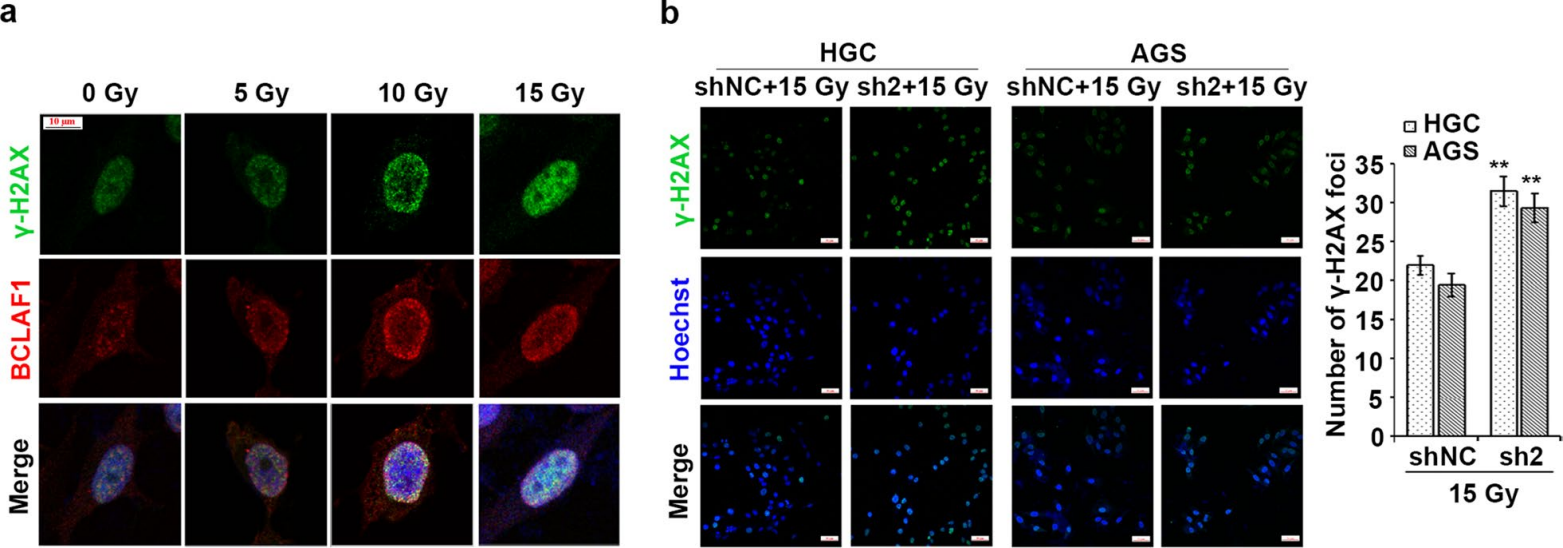
BCLAF1 phosphorylation at Ser290 is involved in DNA damage repair. **a** HGC cells were fixed and stained with indicated antibodies at 2 h after different doses irradiation (0, 5, 10 or 15 Gy). **b** The shNC and sh2 stable cells were treated with IR (15 Gy for 2 h), γ-H2AX level was determined using immunofluorescence. Scale bars, 100 µm. Number of γ-H2AX foci per cell were quantified and statistically analyzed by Student's t-test. **p < 0.01 vs. shNC + 15 Gy

It has been reported that BCLAF1 is a DDR-associated protein which usually form nuclear foci during DNA damage [23]. To determine whether BCLAF1 participates in the DNA damage repair in GC cells, we detected the expression and sub-cellular localization of BCLAF1 after ionizing radiation (IR) exposure. HGC cells were treated with X-rays at different doses and determined BCLAF1 expression by Immunofluorescence. The BCLAF1 and γ-H2AX colocalized in nuclei and the levels of BCLAF1 and γ-H2AX were increased dramatically after IR in a dose-dependent manner (Fig. 6a). As γ-H2AX connotes the existence and degree of DNA damages, and BCLAF1 is reported to colocalize with γ-H2AX foci in nuclei and stabilize the Ku70/DNA-PKcs complex, facilitating non-homologous end joining (NHEJ)-based DNA damage repair [23], to gain an insight into a possible role for BCLAF1 in the DNA damage repair, γ-H2AX foci formation following IR was analyzed by immunofluorescence staining. As shown in Fig. 6b, the γ-H2AX foci formation was almost two-fold higher in BCLAF1 silent cells than in control cells after IR with a dose of 15 Gy at 2 h. The results suggested that BCLAF1 is involved in DNA damage repair and BCLAF1 deficiency delays DNA damage repair and causes cells to maintain a higher level of DNA damage.

**Fig. 7:**
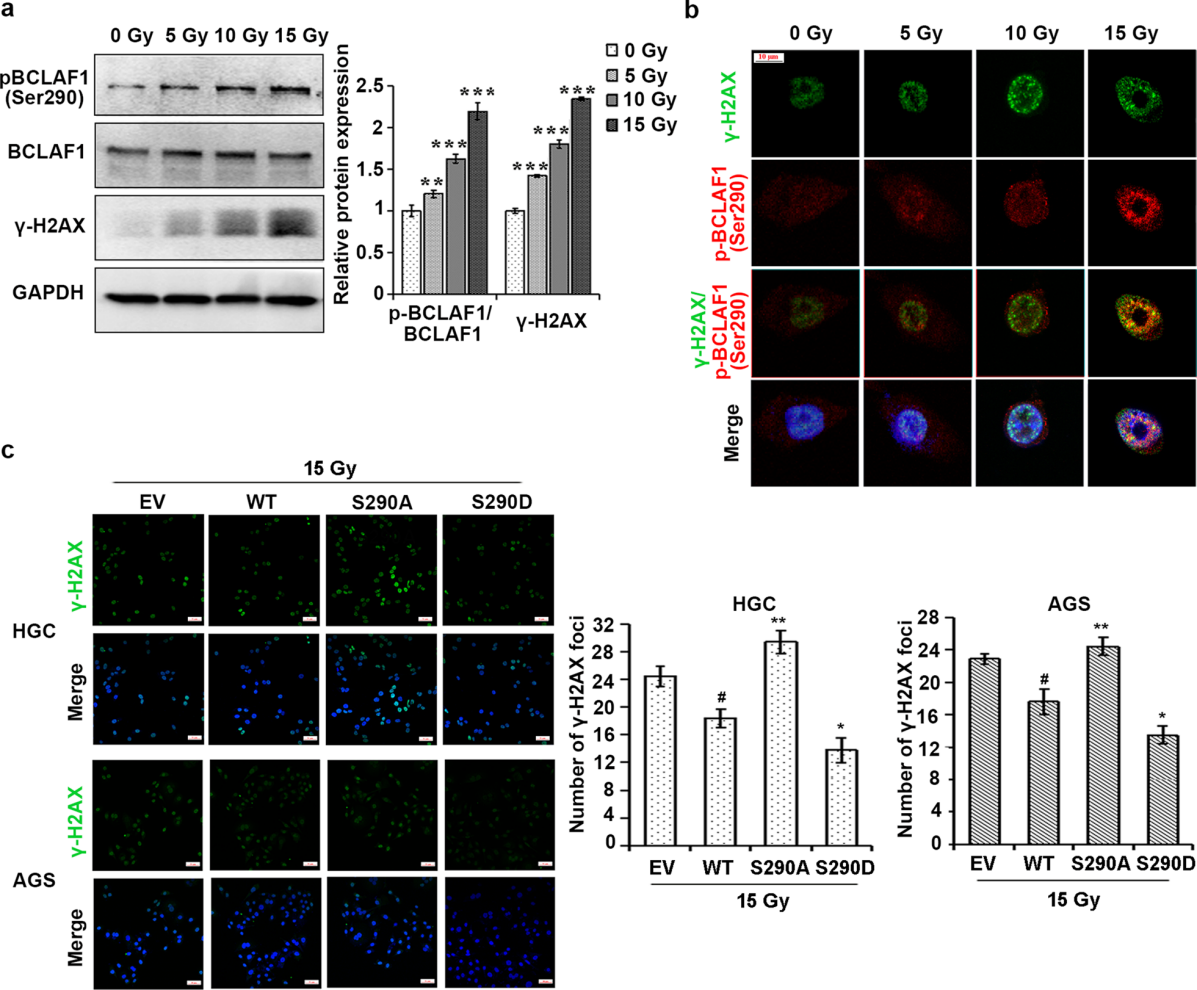
BCLAF1 phosphorylation at Ser290 is involved in DNA damage repair and promotes IR-induced DNA damage responses. **a** HGC cells were treated with 0, 5, 10 or 15 Gy IR for 2 h. Western blot was performed to detect pBCLAF1 (Ser290) and BCLAF1. *p < 0.05, **p < 0.01 and ***p < 0.001 vs. 0 Gy. **b** HGC cells were exposed to IR (0, 5, 10 or 15 Gy) for 2 h. Cells were double-stained with monoclonal antibody to γ-H2AX and polyclonal antibody to pBCLAF1 (Ser290). IR-induced pBCLAF1 (Ser290) foci were colocalized with γ-H2AX foci. Scale bars, 100 µm. **c** HGC and AGS stably cells overexpressing WT, S290D, S290A forms of BCLAF1 were treated with IR (15 Gy for 2 h), γ-H2AX level was determined using immunofluorescence. Scale bars, 100 µm. Numbers of γ-H2AX foci per cell were quantified and statistically analyzed by Student's t-test. *p < 0.05 and **p < 0.01 vs. WT + 15 Gy, ^#^p < 0.05 vs. EV + 15 Gy

We then assessed the importance of Ser290 phosphorylation in the role of BCLAF1 in the DNA damage repair. Western blotting showed that pBCLAF1 (Ser290) level increased after IR besides the increase of BCLAF1 protein level (Fig. 7a). In addition, pBCLAF1 (Ser290) colocalized with γ-H2AX upon irradiation and formed strong IR-induced foci following IR at a dose dependent (Fig. 7b). To further validated the effect of Ser290 phosphorylation in mediating the role of BCLAF1 in the DNA damage repair, HGC and AGS cells were transfected with S290 mutant forms of BCLAF1 (Fig. 7c). Indeed, the overexpression of the non-phosphorylatable S290A mutant led to a significant reduction of DNA damage repair upon IR as determined by the γ-H2AX foci formation. The S290D mutant decreased the radiosensitivity when compared to WT BCLAF1 overexpressing HGC and AGS cells. Our results collectively indicated that BCLAF1 Ser290 phosphorylation is responsible for the role of BCLAF1 in DNA damage repair.

### BCLAF1 phosphorylation at serine 290 protects cancer cells from IR-induced cell apoptosis

**Fig. 8:**
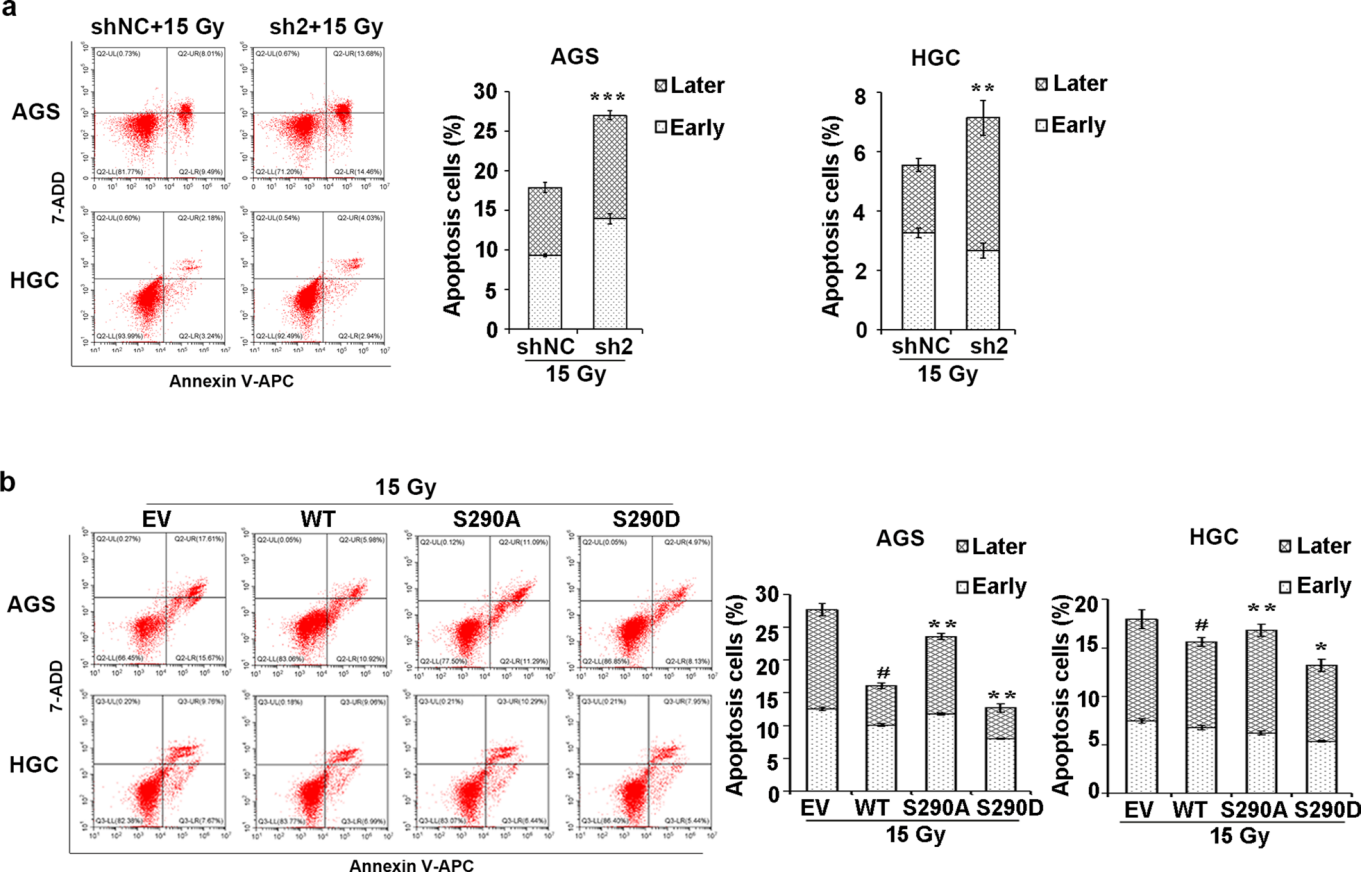
BCLAF1 phosphorylation at Ser290 protects cells from IR-induced cell apoptosis. **a** The shNC and sh2 stable cells were treated with 15 Gy IR for 2 h, and the percentage of cell apoptosis was determined using flow cytometry. Data are shown as mean ± SD of three independent experiments. *p < 0.05, **p < 0.01 and ***p < 0.001 vs. shNC + 15 Gy. **b** Moreover, HGC and AGS cells stably overexpressing WT, S290D, S290A forms of BCLAF1 were treated with 15 Gy IR for 2 h, and the percentage of cell apoptosis was determined using flow cytometry. Data are shown as mean ± SD of three independent experiments. *p < 0.05 and **p < 0.01 vs. WT + 15 Gy, ^#^p < 0.05 vs. EV + 15 Gy based on Student's t-test

Given that BCLAF1 is actively involved in DNA damage repair, the effect of BCLAF1 on IR-induced cell apoptosis was evaluated by flow cytometry analysis at 24 h after 15 Gy X-ray exposure (Fig. 8a). BCLAF1 knockdown cells exhibited significantly more apoptosis than the shNC group post-IR in AGS cells (26.98 ± 1.01% vs 17.85 ± 0.47%, P < 0.001). Similarly, the proportion of apoptotic cells was significantly induced compared with that in the shNC group post-IR in HGC cells (7.15 ± 0.39% vs 5.55 ± 0.12%, P < 0.05), indicating that BCLAF1 knockdown increases cell apoptosis induced by IR. Overall, we found that BCLAF1 knockdown cells showed a reduction in cell proliferation and an elevated basal γ-H2AX foci and higher susceptible to IR-induced DNA damages and cell apoptosis, suggesting that BCLAF1 expression might affect the radiosensitivity of GC cells.

We further validated the effect of Ser290 phosphorylation of BCLAF1 on IR-induced apoptosis. AGS and HGC cells that stably expressed WT, S290D, or S290A forms of BCLAF1 were treated with 15 Gy X-ray. We collected each group of cells for apoptosis analyses (Fig. 8b). AGS and HGC cells with WT BCLAF1 overexpression were more resistant to IR than empty vector cells. Moreover, we found that substitution of Ser290 with A or D led to the promotion or inhibition of apoptosis in AGS and HGC cells post-IR. In conclusion, these results indicated that the phospho-deficiency of BCLAF1 Ser290 enhances IR-induced DNA damage and apoptosis, suggesting that the Ser290 phosphorylation is responsible for the function of BCLAF1 on the radiosensitivity of GC cells.

## Discussion

In the present study, a simple open search method without pre-enrichment of phosphorylated proteins is used in our work and the method may be applied for different protein modifications. 832 unique phosphorylated sites spreading over 382 proteins were identified. As described in this manuscript, the up-regulated protein phosphorylation identified by this approach is demonstrated to regulate DNA replication, DNA repair and mRNA splicing, which consistent with the research explored by Tong et al. [28]. In general, it can be a convenient and rapid method for the discovery of new protein modifications and biomarkers for different cancers.

In comparison with para-cancerous tissues, the phosphorylation of Ser290 at BCLAF1, a DNA damage response-associated protein, is the mostly upregulated in GC tissues. BCLAF1 was a widely expressed gene that encodes a protein with homology to the basic zipper and Myb DNA binding domains [29]. Studies have indicated that BCLAF1 involves in diverse biological processes, such as DNA damage repair, cell proliferation and angiogenesis [14–16, 23]. Consistent with these reports, our results indicated that BCLAF1 knockdown reduced cell proliferation and increased cell susceptible to IR-induced DNA damages in GC cells. Recent studies indicated that BCLAF1 function mainly depends on its phosphorylation, for example, Ewald Heroes et al. reported that the BCLAF1:SDS22 interaction is dependent on the phosphorylation of BCLAF1 [30].

By searching the uniport protein database, 46 phosphorylation sites were found in BCLAF1, but the significance and function of these sites have not been reported. In this manuscript, we firstly found that level of pBCLAF1 (Ser290) in GC tissues is significantly increased and can be used to predict poor survival outcome for GC patients, indicating that pBCLAF1 (Ser290) may play an important role in GC. Therefore, clarifying how pBCLAF1 (Ser290) regulates GC has important implications for understanding the pathogenesis and progression of GC. In GC cells, we showed that pBCLAF1 (Ser290) was increased upon IR expose and promoted DNA damage repair and protected cells from IR-induced cell apoptosis. Thus, our results indicated that Ser290 phosphorylation is one of the critical forms of BCLAF1 modification in regulation its function in GC.

Our understanding of DNA damage response provides a new approach to disease management [31, 32]. The protein H2AX is rapidly phosphorylated at the serine 139 site (γ-H2AX) in response to extensive DNA damage [33]. γ-H2AX induction is one of the earliest events in DNA damage response and plays an important role in the perception and repair of DNA damage [34] by promoting stable accumulation of many other signaling and DNA repair proteins including 53BP1 [35], GADD45A [36] and BRCA1 [37] at DSB sites. Particularly, BCLAF1 is an IR-induced H2AX-interacting partner involved in γ-H2AX-mediated regulation of DNA damage repair and cell apoptosis [23].

Our data confirmed that IR induced the phosphorylation and co-localization of BCLAF1 and γ-H2AX. Moreover, mimic and deficient Ser290 phosphorylation in BCLAF1 significantly affected the DDR and IR-induced apoptosis. These indicated the significance of Ser290 phosphorylation in the involvement of BCLAF1 in DDR and subsequent cancer cell apoptosis. However, the underlying mechanisms of how IR increase the pBCLAF1 (Ser290) and how this phosphorylated BCLAF1 is recruited to the DNA damage loci need further investigation. It will be valuable to have future studies include what proteins associate with BLCAF1 Ser290 phosphorylation and help derive a mechanism of how this phosphorylation event works in a radiosensitive manner.

## Conclusions

Taken together, we reported a serial of GC-associated phosphoproteins, further identification and analysis of these protein modifications will expand our understanding of GC. The identification of the upregulation of pBCLAF1(Ser290) suggested a potential biomarker for the prognosis of patients and a possible target to improve radiotherapy sensitivity in GC.

## Supplementary Information

**Additional file 1: Figure S1.** Plasma cells were used as the negative control.

**Additional file 2: Figure S2.** A diagram of the sequential steps used in completing the proteomics experiment.

**Additional file 3: Figure S3.** Western blotting of the lysate pretreated with CIP (phosphatase).

**Additional file 4: Table S1.** Additional table.

## Data Availability

The datasets used and/or analysed during the current study are available from the corresponding author on reasonable request.

## Abbreviations

BCA: Bicinchoninic acid
BCLAF1: Bcl-2-associated transcription factor 1
BSA: Bovine serum albumin
DDR: DNA damage response
DMEM: Dulbecco’s modified Eagle’s medium
EdU: 5-Ethynyl-2′-deoxyuridine
FBS: Fetal bovine serum
FDR: False discovery rate
GC: Gastric cancer
γ-H2AX: Phospho-histone H2AX
HPLC: High performance liquid chromatography
IAA: Iodoacetamide
IR: Ionizing radiation
LC–MS/MS: Label-free liquid chromatography-tandem mass spectrometry
OD: Optical density
PTM: Post-translational modification
PVDF: Polyvinylidene difluoride membranes
SAAPs: Single amino acid polymorphisms
SD: Standard deviation
Ser290: Serine 290
TFA: Trifluoroacetic acid
TMA: Tissue microarray
UA: Urea
