# Supplementary figures and images for: Bcl-2-associated transcription factor 1 Ser290 phosphorylation mediates DNA damage response and regulates radiosensitivity in gastric cancer
Source: J Transl Med. 2021 Aug 9;19:339. doi: 10.1186/s12967-021-03004-z (PMC8351323; doi:10.1186/s12967-021-03004-z)

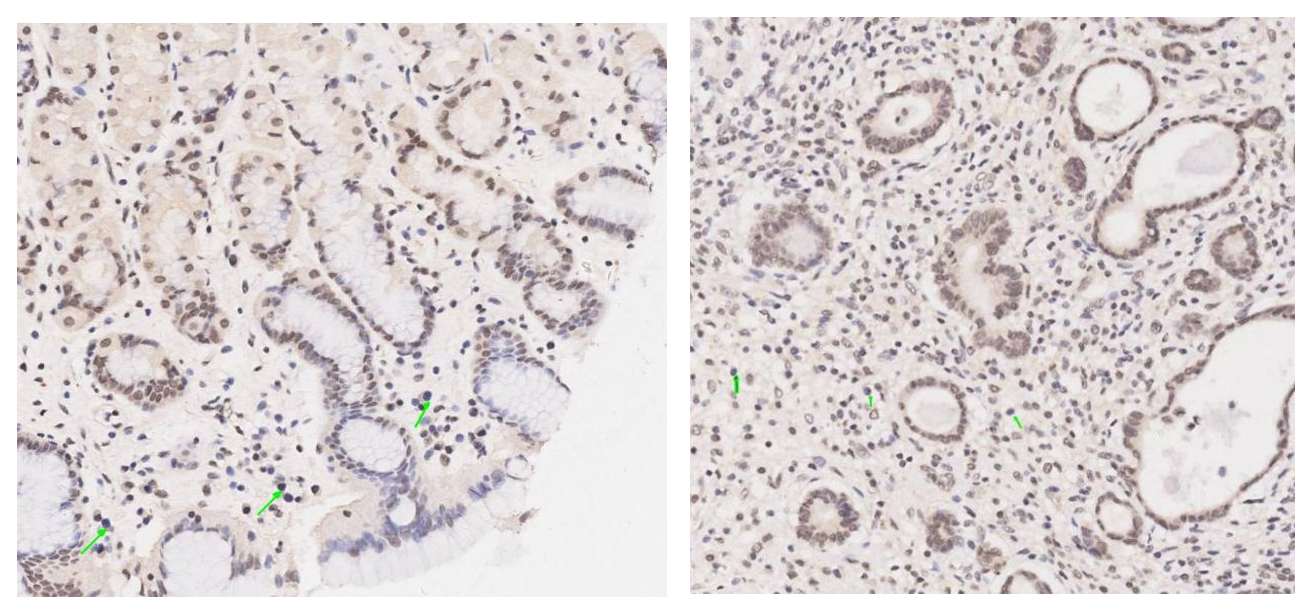

Supplement: Supplementary file 1 — Additional file 1: Figure S1. Plasma cells were used as the negative control. [file 12967_2021_3004_MOESM1_ESM.tif]

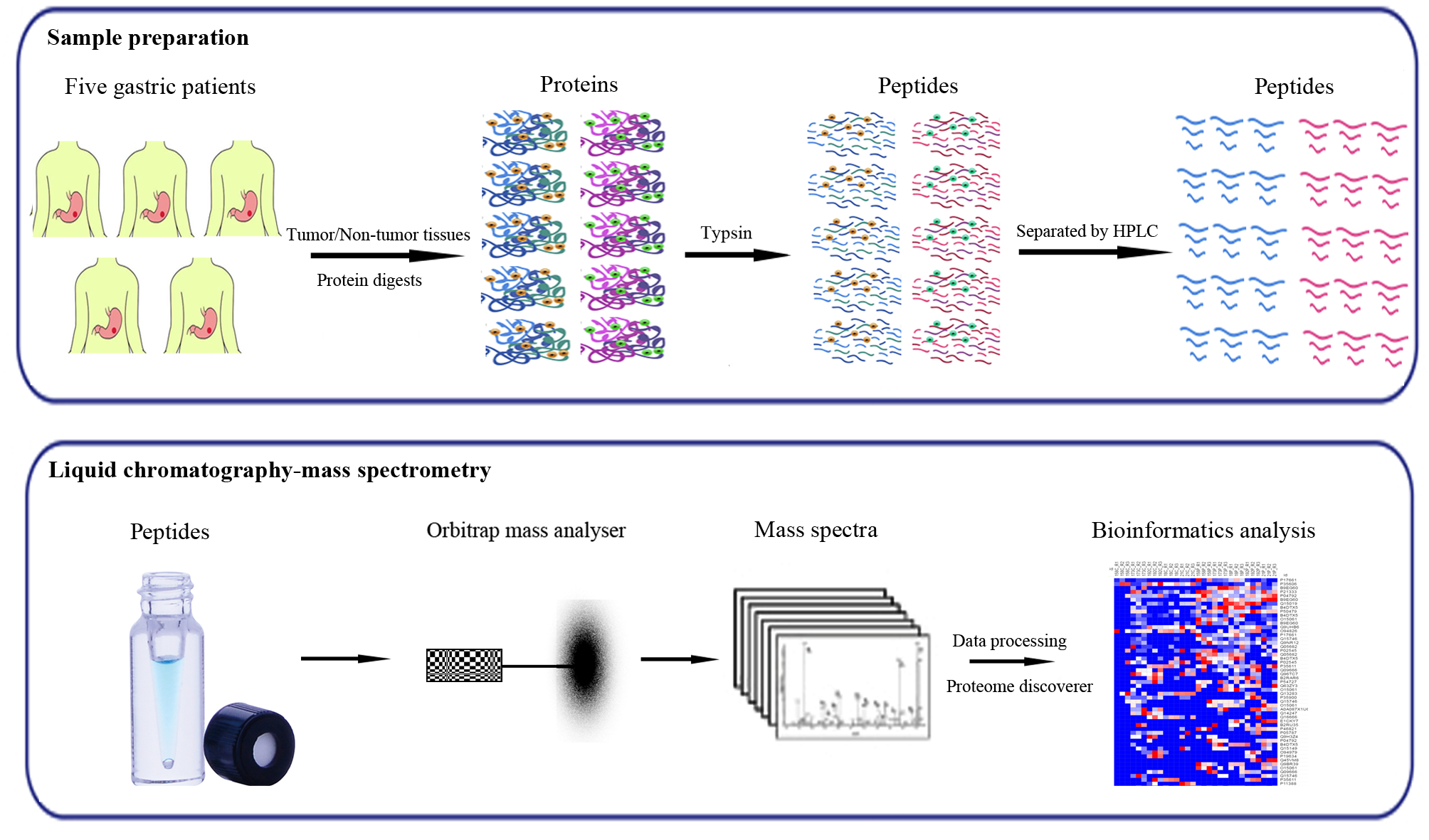

Supplement: Supplementary file 2 — Additional file 2: Figure S2. A diagram of the sequential steps used in completing the proteomics experiment. [file 12967_2021_3004_MOESM2_ESM.tif]

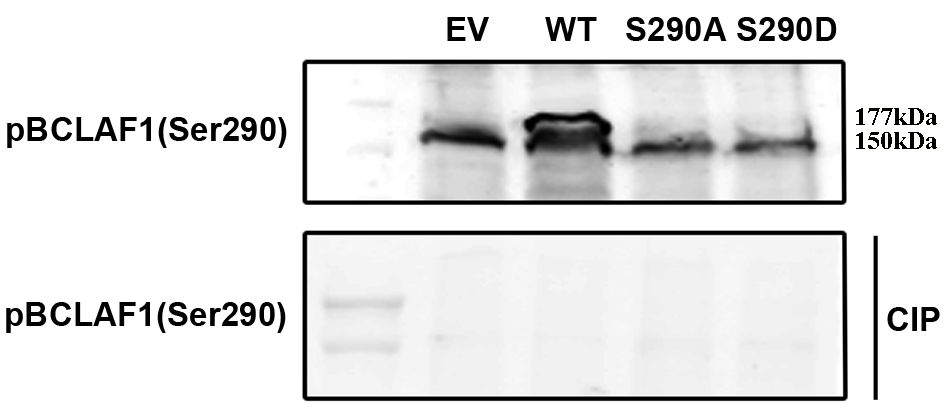

Supplement: Supplementary file 3 — Additional file 3: Figure S3. Western blotting of the lysate pretreated with CIP (phosphatase). [file 12967_2021_3004_MOESM3_ESM.tif]
